# Broadening diversity through creative involvement to identify research priorities

**DOI:** 10.1186/s40900-020-00244-z

**Published:** 2021-01-06

**Authors:** Stephanie Tierney, Shoba Dawson, Anne-Marie Boylan, Gillian Richards, Sophie Park, Amadea Turk, Opeyemi Babatunde

**Affiliations:** 1 Nuffield Department of Primary Care Health Sciences, University of Oxford, Oxford, UK; 2 Centre for Academic Primary Care, Population Health Sciences, Bristol Medical School, University of Bristol, Bristol, UK; 3 Department of Primary care and Population Health, UCL, London, UK; 4 School of Medicine, Keele University, Staffordshire, UK

**Keywords:** Art, South Asian heritage, Creative approaches, Dementia, Diversity, Mental health, Research prioritisation

## Abstract

**Background:**

Patient and public involvement (PPI) can help with steering and shaping research prioritisation and execution. However, some groups of people may not be encouraged to take part and their voices may be seldom listened to in the production of research. This is important to consider because they may have poorer healthcare experiences. We tried using art as a vehicle for including individuals not necessarily invited to be part of research priority setting.

**Methods:**

We contacted existing groups and organisations to reach people not routinely supported to be part of PPI. We targeted individuals: a) with dementia, b) with a mental and physical health condition, c) of South Asian heritage. We ran a workshop with each group at which individuals shared their experiences of healthcare. A young amateur artist also attended, who produced a piece of artwork afterwards that reflected the research priorities raised. We held a Twitter chat to discuss these pieces of art and the processes involved in their generation.

**Results:**

From each workshop, we produced a list of research priorities. These included: a) improving coordination of care for people with dementia, b) information needs and anxiety/guilt around accessing care for people with physical and mental health conditions, c) supporting discussion of women’s health issues in South Asian communities. These priorities were reflected in three pieces of art, which can be viewed online. Feedback from those at workshops suggested that the artwork helped them to feel that their voice had been heard and triggered their interest in how research is developed. Those involved in the Twitter chat commented that art was one means through which researchers could connect with a range of groups in a PPI context when preparing and producing a study.

**Conclusions:**

We found the medium of art to be an effective way of including a range of people in research prioritisation setting. This approach could be useful for future PPI, building on what we have learnt from the project described in this paper.

## Plain English summary

Patient and public involvement (PPI) in research has become more prominent in recent years. Drivers for incorporating the voices of lay people into the planning and conduct of research include: a) to ensure that research addresses issues of importance to patients, b) informing how public funding is spent, and c) helping to design a study in a way that is acceptable to potential PPI contributors. There are specific groups that may be defined as ‘seldom listened to’ when it comes to PPI activities. We were interested in how to engage such populations in setting priorities for research. In particular, we sought to work with people: a) living with dementia, b) affected by co-occurring physical and mental health conditions, c) of South Asian heritage. We used the creation of artwork as a vehicle for broadening diversity in the setting of priorities for research. We held separate workshops with individuals from each of the groups listed above. They were invited to talk about their health/care needs, experiences of services, and to identify topics where future research should focus. An artist attended each group. They produced some artwork based on discussions around research priorities. We have shared this artwork with workshop attendees, and more widely through a blog, a narrated video and a Twitter chat. In this paper, we present our reflections on undertaking this project, lessons learnt and how this art-based approach could be used by others going forward.

## Background

Debates are ongoing about how to incorporate a diverse range of voices in the design and delivery of research as part of patient and public involvement (PPI) [1–4]. They highlight a lack of diversity and inclusion in PPI activities of people from varying ethnic backgrounds, and limited representation of those living in socially and economically disadvantaged areas, or absence of those with a cognitive impairment or with a mental health condition [5, 6]. Their missing input into the planning and conduct of research is unfortunate as these populations can have relatively poor health outcomes (e.g. morbidity, mortality) and may encounter negative experiences of services [7, 8]. Their involvement in research can be beneficial, ensuring that it addresses issues of interest to key stakeholders and improves the relevance of research questions to practice and policy [9]. Therefore, reaching beyond the ‘usual suspects’ (often defined as older, White, middle class) who take part in PPI activities [10] is important. A diversity of voices in PPI means that research will be shaped by a plurality of views and perspectives. Ensuring equity, diversity and inclusion in research planning and execution involves thinking about things like payment, language used and how information is communicated, as well as accessibility of places where meetings are conducted and the way in which interactions with PPI contributors are structured and performed [11, 12].

Although traditional approaches to PPI (e.g. formal meetings) are useful, they may structurally limit who takes part. Hence, exploring innovative ways to facilitate PPI is required to allow for dialogue and engagement with a broader range of people. This includes producing novel approaches to co-producing ideas for research prioritisation (i.e. topics that warrant developing into proposals to be submitted for research funding). Art can stimulate discussion in an accessible manner [13]; it is used across cultures and settings as a form of communication that can foster responses such as awareness, contemplation and a drive to react [14]. A review of arts-based research in health highlighted how it can access alternative ways of knowing and is a way of making research accessible to a wide audience [15].

We wanted to explore whether an arts-based approach to PPI could be a vehicle to support the inclusion of individuals who are not usually invited to identify priorities for research. We tried to ensure that the environment for accessing their views was relaxed and thought that a focus on producing some artwork would make this a less formalised approach compared to a more structured meeting in a university based environment. Consequently, we used the development of artwork, following group discussions and priority setting, to a) encourage groups to be involved, b) provide an output that showed a group’s views had been listened to, c) act as a mechanism to inspire further discussion as part of PPI, d) provide researchers with a visual indication of the research priorities identified by each group.

## Aim

The project aimed to explore the use of art as part of PPI research priority setting with groups who are seldom listened to in this process. We outline a series of involvement activities that we undertook to provide a voice to people with dementia, people with co-occurring physical and mental health conditions, and people of South Asian heritage. A PPI contributor (GR) was part of the project team; she helped with developing the idea for this project. We offer reflections on our experiences, lessons learnt and how this approach could be used by others going forward to identify and work with seldom listened to groups.

## Methods

### Groups involved and settings

As the intention was to broaden the reach of PPI, this project involved a multisite collaboration that consisted of workshops with three different populations:

- In Oxford - people with young onset dementia;
- In Bristol - women of South Asian heritage.
- In Keele - people with musculoskeletal (MSK) and mental health problems;

We approached community groups to locate individuals meeting the characterises above who had not been involved in PPI research activities previously. One of the researchers (SD) had an existing relationship with the organisers of the community group attended by women of South Asian heritage, but not with those taking part in the workshop. In this paper, we use the term PPI contributors to refer to individuals who engaged in research priority setting for the project. None of the project team knew them in advance of this work.

### Artists and art involvement

At the outset, we planned to explore the involvement of amateur artists. The team thought it would be a good opportunity for young people with an artistic flair to develop their portfolio and to engage in a project that was likely to have national coverage. In addition, it was an opportunity for them to learn about health services research and PPI. We recruited young people through local advertisements and invitations via schoolteachers to students (at secondary school or college) to be part of the project. Two young amateur artists (E. Prior (EP) aged 17 and M. Viljoen (MV) aged 19) responded and were part of the team on this project. They worked independently of each other. MV attended workshops in Bristol and Keele, whilst EP was at the Oxford workshop. Artists produced their artwork after attending a meeting, where they listened to discussions, asked questions and made notes. Furthermore, they had follow-up conversations about their ideas with the researcher who was also present at a meeting. They were provided with art materials, £50 for their contribution to the project, and their travel expenses were reimbursed. Artists came with a relative naïve understanding of the topics being explored. They were supported by researchers who had conducted studies in these broad areas of dementia, mental health and research involving people of South Asian heritage.

### PPI workshops

At each workshop, those attending were invited to share experiences of their health/care needs, services, and to identify priorities for research. We also highlighted what is meant by PPI in research, and how people might participate in this activity. In addition, we asked for suggestions about how best to share findings from this project with a diverse audience. All workshops took place in person during February 2020. Notes were made by researchers to record key points that were raised. Attendees of the workshop received a high street gift token to thank them for their involvement.

In Oxford, ST attended a young onset dementia group that meets once a month for 2 hours in a local pub during the day. She had 50 min to talk to the group, which on the day consisted of 8 people (5 with young onset dementia, 2 relatives and the group coordinator).

SD contacted an organisation in Bristol; she knew its organisers from a previous piece of research, but not for PPI purposes. She attended a meeting that this group was holding at a local community centre during the day. She facilitated a 70 min discussion with 11 women of South Asian heritage. SD communicated in Hindi as women who were present understood this language. An interpreter was also present to support discussions in Punjabi and to ensure that information was not lost or misinterpreted because of language differences. SD and the interpreter backtranslated the information in English to ensure that the artist could take notes.

In Keele, OB approached the College of the Third Age as a vehicle for recruiting older people with MSK and mental health problems. A date was arranged for those who were interested to attend a meeting at the University. Lunch was provided beforehand. Six older adults with various chronic MSK problems (e.g. back pain, arthritis, fibromyalgia) and mental health conditions (depression and/or anxiety) were present. 150 min were spent discussing lived experiences of their conditions, care and priorities for research.

**Fig. 1:**
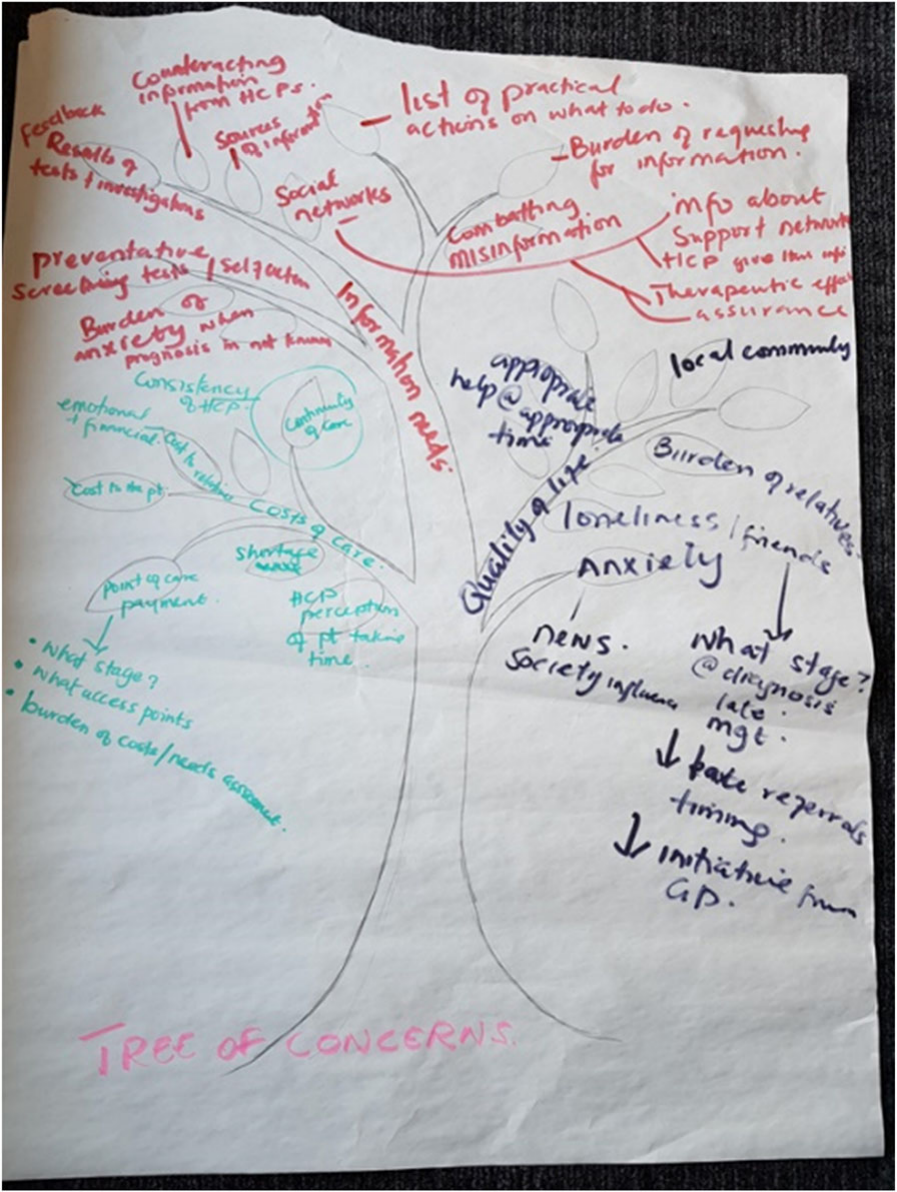
Tree of concerns created in the workshop at Keele

At each workshop, those attending were invited to prioritise ideas for research that they had produced from an initial, free-flowing discussion. In Bristol, topics that might be of interest were discussed in advance with the community group organiser. At the workshop, these topics were presented to the group to arrive at a consensus on one that they wanted to discuss (and this became the focus of the artwork produced from this group). In Oxford, topics were written by the researcher on a post-it note as they arose during the initial discussion (which lasted for about 35 min). Attendees were then given three sticky dots and asked to place them on topics that were of most importance to them. In Keele, a tree of key concerns was created by the group to identify and represent their research priorities (see Fig. 1).

An artist was present at each workshop to listen and ask questions of the group. They made rough sketches and noted topics of interest to be captured in the final artwork. Afterwards, researchers had a debrief with each artist, to explore initial ideas and reflections. Subsequently, the artists produced pieces of art based on priorities for research identified by each group. Draft artwork was shared electronically with groups to ensure that it reflected their expressed concerns and priorities. Feedback was positive in this respect from group members.

## Results

### Research priorities

Research priorities to transpire from each PPI workshop are listed in Table 1. Engagement with the young onset dementia group at Oxford brought to light the need for evidence on coordinated support post diagnosis, so that people given this diagnosis can be helped to live as full a life as possible. At Bristol, South Asian women highlighted the hidden nature, myths and rumours associated with women’s health issues, specifically menstruation. Their conversations indicated a need for research into the lack of informed knowledge, and the difficulty of discussing women’s health issues openly and in the presence of male members of the community. At Keele, concerns and key challenges of people with MSK and mental health problems that were raised included the burden of feeling isolated, the sometimes unhelpful treatment given for multiple conditions, finding the right information from the internet or other sources, and the “guilt/fear” of being judged as wasting health professionals’ time. Hence, three research priorities for this group centred on information needs, quality of life/anxiety, and cost of care.

**Table 1:** Research priorities identified during group discussions

Young onset dementia	South Asian women	MSK and mental health
How to ensure everyone has access to a range of support after being diagnosed with dementia?	What are the barriers and enablers to communication around menstruation and menopause?	How to address lack of information related to accessing health care (lack of navigation, inadequate signposting, and lack of feedback for example following test results)? How to address the risk of misinformation from the internet or other sources (social networks, self-diagnosis)?
How to improve the coordination of services/support for people with dementia (and their family)?	How can we educate and support women from South Asian communities to engage in discussions around menstruation and menopause?	Due to limited consultation time with healthcare professionals, how do we reduce the burden of finding the right information for self-management as and when needed?
How to support people with dementia to engage in positive health behaviours (e.g. a good diet, getting vaccinated for flu, keeping physically active)?		How to combat loneliness, and isolation when living with multiple physical and mental health conditions?
How to best educate people about the condition (the general public, healthcare professionals, people living with dementia)?		How to manage anxieties, “guilt/fear” of being judged as wasting health professionals’ time at consultations?
How to address the employment challenges and potential discrimination in the workplace that people with young onset dementia can encounter?		How to minimise cost and address the burden of uncoordinated care and treatments for multiple physical and mental health conditions?

Table 2 also highlights some overarching issues that cut across all three groups, in terms of having access to equitable care, improving health communication and supporting health literacy.

**Table 2:** Learning point 1 – regular communication

If replicating this work, regular communication with the artist(s) is critical. Set up an initial meeting to discuss what being part of a project involves. Give them time to ask questions. Be open and accessible as researchers. Encourage the artist to express any concerns or queries. Offer feedback and constructive suggestions as artists sometimes questioned whether what they were producing was what the project team required or expected. We would extend this importance of regular communication to the groups involved in workshops, so they understand what the project entails, how they can contribute and are provided with access to the final artwork. We also, as a team, met once a month to ensure that the work being carried out in different settings was in line with the project’s aim, budget and deadlines.	

### What we have learnt from undertaking the project

Involving groups with the end goal of producing some artwork was effective in the following ways. Firstly, it encouraged people not ordinarily asked about research priorities to voice their opinions, which then shaped a piece of artwork. This allowed us to explore concerns based on their experiential expertise that could inform and underpin future research. Secondly, the end products – the artwork – offered an eye-catching and accessible way to disseminate these potential ideas for future research.

People involved were motivated by the prospect of helping to produce some artwork; they liked the idea of contributing to this through sharing their views and experiences. We sent them a photograph of the finished artwork produced from their group discussion. We asked for their thoughts on this and on the research priority it reflected. Their feedback stressed the power of images created by the artists, which distilled their experiences. They noted how they welcomed the opportunity to share their views, and to have these turned into ideas for potential research projects. Hence, the use of art was an effective channel for reflecting the views of these groups in terms of research priorities. It allowed the project team to present and disseminate these priorities in a visual format.

### What we feel went well with the project

**Fig. 2:**
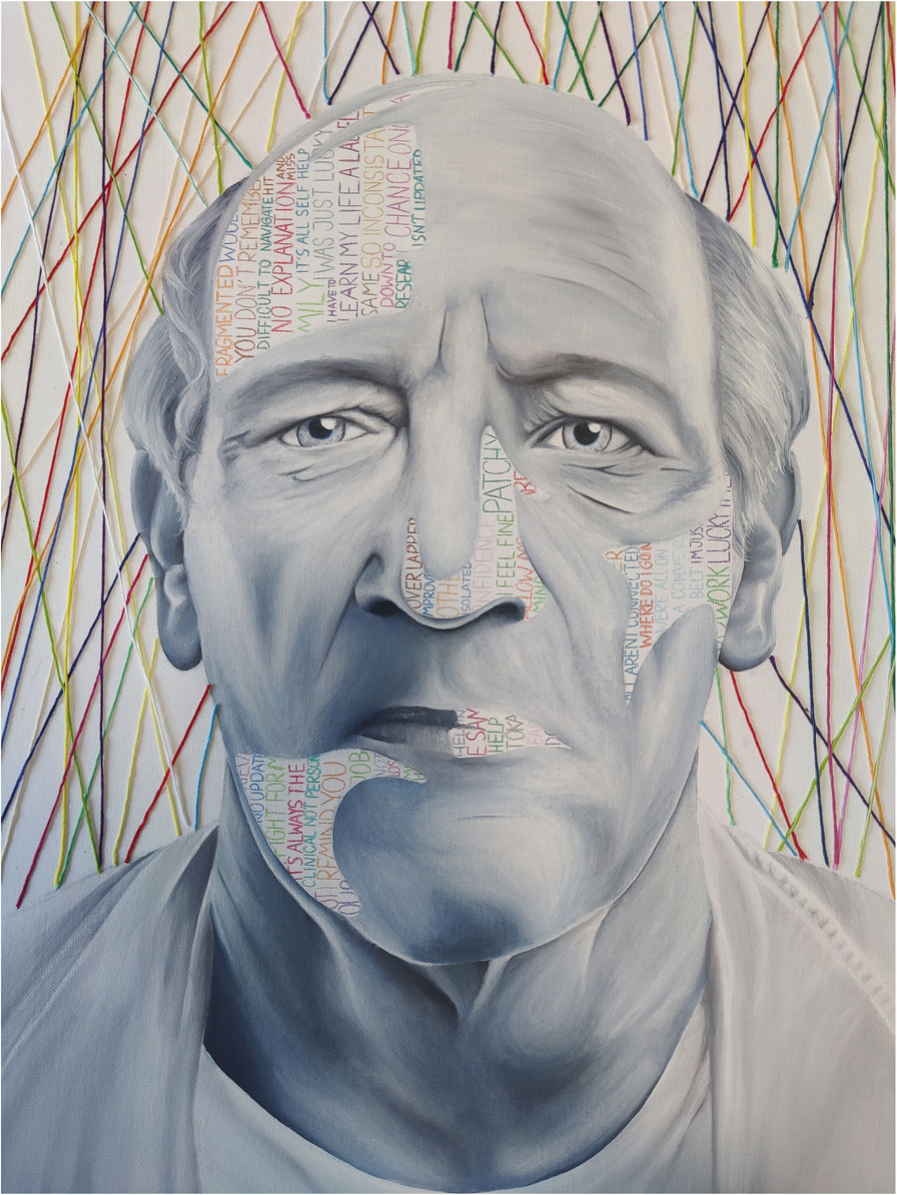
Untangling dementia: Understanding personal, social and structural influences on experiences of dementia to develop priorities for research

**Fig. 3:**
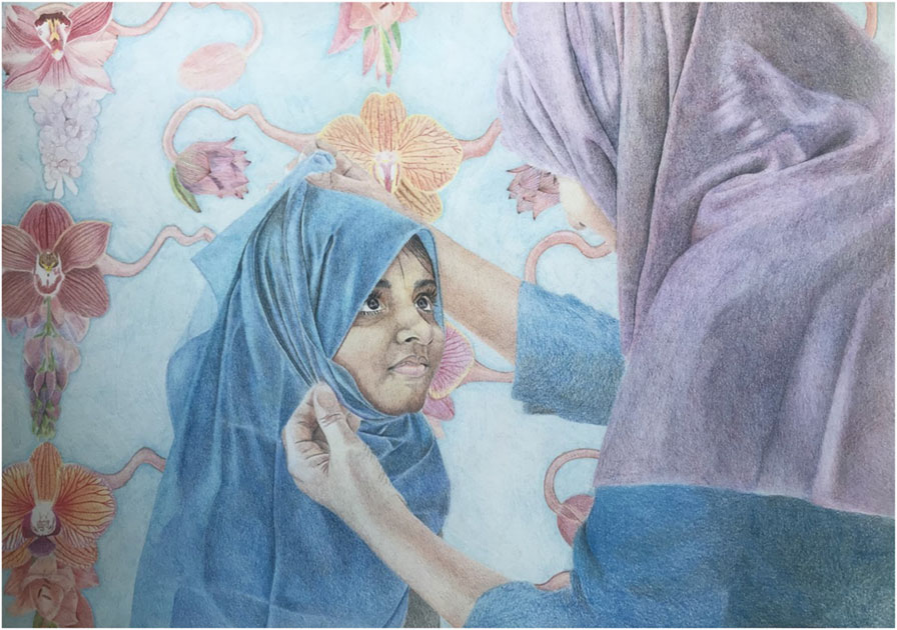
Meanings of menstruation and menopause: Cultural influences on management and experiences

**Fig. 4:**
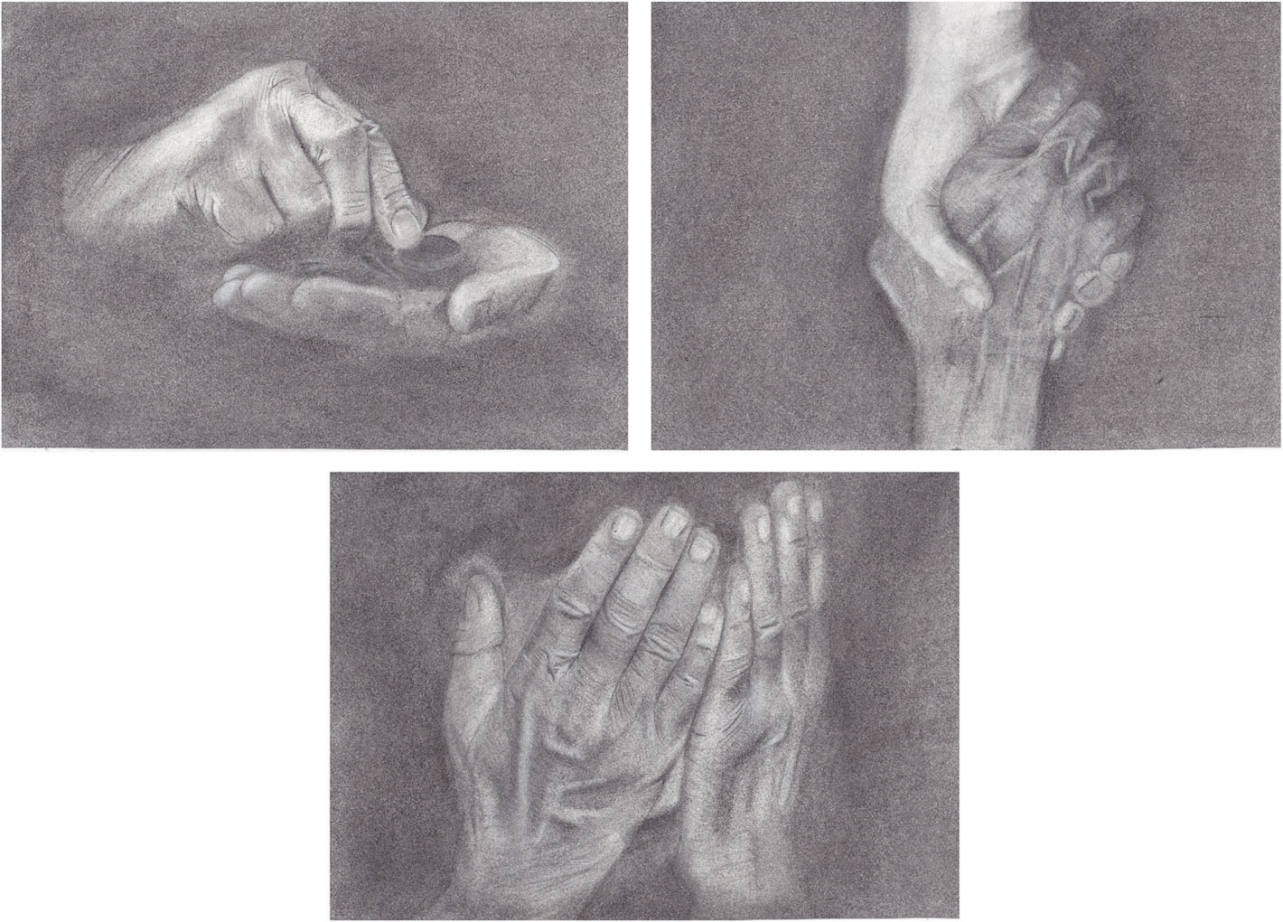
The trilogy of hands: Healthcare priorities for older adults with physical and mental health conditions

Working with the young artists was a positive experience. They were reliable in terms of attending meetings, providing us with updates, and asking for feedback. They produced powerful pieces of art, which are displayed below (see Figs. 2, 3 and 4) and can be viewed online [16]. We have also created a narrated video of the final artwork, with a description of what each piece reflects (www.spcr.nihr.ac.uk/news/blog/using-art-to-engage-with-people).

We asked each artist to provide a brief written reflection of how they found their involvement in the project. They appreciated having the opportunity to learn about the experiences of people from different backgrounds; this exposed them to issues that they did not face in their personal day to day life. It also introduced them to the research process. In conversations with these young artists about their role in the project, they expressed some initial concerns about being able to produce the type of artwork that we, as researchers, were expecting. They also said they had some apprehension about being able to reflect group discussions adequately or about offending people because they did not share the same culture. However, they commented that any concerns were allayed through regular communication with the researchers; we supported the artists via regular emails and telephone calls, to ensure that they were happy with what they were doing (see Table 2 – learning point 1). This is reflected in the following feedback from one of them:

*“I feel that the organisation of the project went well … communication was good … My artwork hopefully conveys the key priorities … I have also been able to weave some of this project into my A Level, which adds another dimension to my coursework.” (E. Prior)*

Both artists said they would work on a similar project if asked again:

*“Yes, I have thought about issues that I wouldn’t in my everyday life and I have gained empathy for different walks of life.” (M.Viljoen).*

### What we feel could have gone better

Initially, we struggled to find young artists to work with us (see Table 3 – learning point 2). Over the first months of the project, we contacted several colleges and schools but were unable to get past gatekeepers to invite students to be artists. We sent several emails and, in some cases, made telephone calls, without success. We eventually used work contacts, sending out a request to colleagues asking if they knew of any young people (family members or friends) who might wish to be involved. We invited potential artists to share their work with us. If we were happy with their artistic skills, we met them in person or by phone to talk about the project and what it would involve, so they had a good understanding before committing to work with us.

**Table 3:** Learning point 2 – locating artists

Factor in sufficient time for locating an artist. Think about using existing contacts (e.g. colleagues, school governors). Consider alternative recruitment routes to schools/colleges (e.g. youth parliament, young people’s involvement group at a local hospital, youth groups, church groups, outreach workers).	

PPI workshops took place just before the COVID-19 pandemic hit the UK and lockdown ensued. At the workshops, we discussed with PPI contributors how we might best disseminate the artwork beyond academics (e.g. to those who might be similar to the groups involved). Workshop attendees suggested this might include sending short summaries to existing groups (e.g. Young Dementia Network, College of the 3rd Age) for them to publicise (e.g. in newsletters). Attending meetings in person to talk about the project was also proposed, so that people could ask questions. This was stalled to an extent by social distancing rules.

### Thoughts about what we would have done differently

The groups in Oxford and Bristol were pre-existing; hence, researchers attended one of their regular meetings. Attending a venue and time when groups usually meet was beneficial in terms of not having to arrange a separate meeting and ensuring that there was good attendance. In addition, because individuals knew each other, and were in a familiar environment, they were comfortable discussing their experiences together. However, it meant that the researcher had less control over how long they had to talk to the group; if there were other items on an agenda this reduced the time available to discuss research priorities. In addition, the setting may not necessarily be conducive to groupwork; for example, the young onset dementia group met in a local pub, which although quiet, made group activities more difficult (see Table 4).

**Table 4:** Learning point 3 – using existing groups

Attaching the workshop to an existing group meeting facilitated set up and meant that those attending were happy to talk as they already knew each other. It did limit the control a researcher had in terms of time devoted to discussing research concerns or in setting up activities. Nevertheless, it is important to consider whether meeting in a venue arranged by researchers may mean that the needs of the team overshadow those whose views are seldom listened to, if individuals feel more comfortable talking in a familiar setting at a familiar time. This may have been the case especially for those with young onset dementia. This highlights the issue of power relations in PPI. There is sometimes an expectation that PPI contributors will ‘come to researchers’, whereby we ask individuals to fit with the setting, communication, approaches with which we, as researchers, are familiar. This is likely to exclude certain people. Democratising and distributing spaces in which we interact as part of PPI is, therefore, important. This may be encountered as inconvenient to researchers’ norms. However, overall, it can be productive and positive, enabling new and diverse groups to participate.	

At each meeting, one of the artists sat in the room, listened to the discussions and interacted with the attendees. They then used this interaction and the notes they and the researchers made to create a piece of artwork over the following month that reflected the priority selected by the group on the day. We had discussed as a team co-producing the artwork more closely with the groups (either getting them to share their own sketches or concepts for the artwork with the artist or inviting them to produce something with the artist over several meetings). In the end we decided to keep this first attempt at using artwork to reflect seldom heard groups’ research priorities relatively simple. Therefore, we asked the artist to attend and engage in the meeting, and then to form the artwork outside of the workshop.

### Sharing what we have done and learnt

Alongside the production of a blog [17], our narrated video (see above), and this paper, we are sharing the finished artwork with each group that was involved. An online meeting of the young onset dementia group was attended by ST, at which she showed those present (*n* = 8) the final picture. This led to a frank discussion about what it meant to them and how it reflected their own lived experiences in terms of the emotions and struggles encountered post diagnosis (including losing a job, social contacts reducing and poor coordination of support).

Further face-to-face communication of findings is planned in Keele and Bristol – depending on social distancing rules due to COVID-19. Meeting face-to-face is particularly important for the Bristol group, given that those present were not fluent in speaking or reading English. The community group organisers were contacted, and findings were shared with them. Their input was sought to ensure that the artwork and accompanying text was depicted in a culturally sensitive manner, and that information was not lost in translation. The organisers for this group at Bristol, and people attending the Keele workshop, expressed a desire for and gave researchers an open invitation to engage in further discussion and dissemination of findings to wider networks in the future.

To support dissemination, we held a Twitter chat to share our work and to learn from others’ experiences of using similar arts-based approaches. Twitter chats are public conversations that take place on a specific topic through this social media platform. Usually, these discussions are moderated, and take place at a designated time with a predetermined hashtag. Anyone wishing to engage posts their comments or answers questions by including the hashtag as this allows everyone to follow the conversation. The purpose of the Twitter chat was to engage with patients, public members, researchers and healthcare professionals about our research, to share our work and to learn from their experience of being involved or undertaking something similar.

**Fig. 5:**
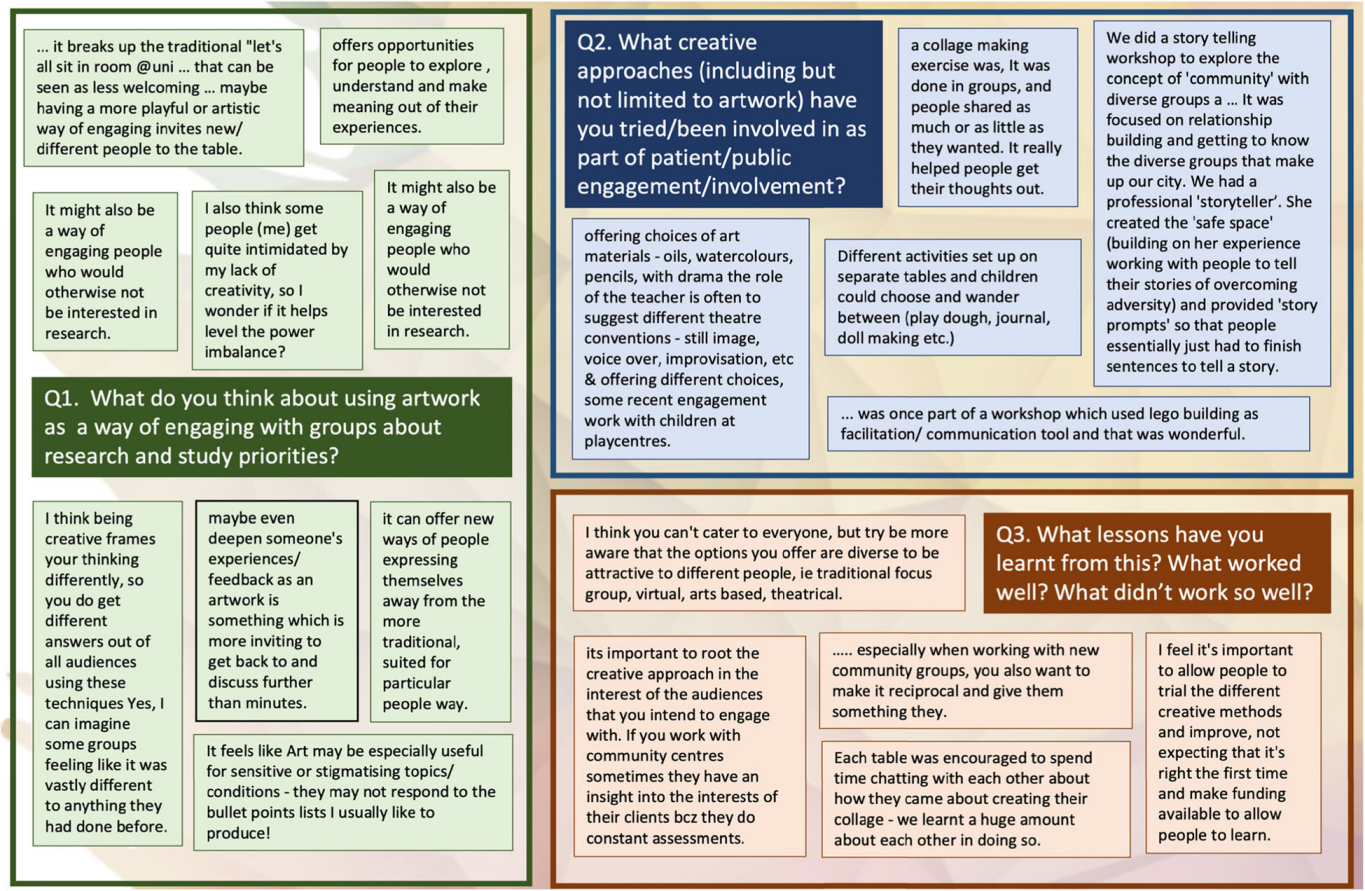
A snapshot of discussions on the Twitter chat

We advertised the Twitter chat a month in advance and sent an email about it to researchers, PPI contributors and coordinators, and other relevant organisations that might be interested. The Twitter chat involved an hour dedicated to discussing online the following questions: a) using artwork as a way to engage with groups about research priorities; b) creative approaches people had tried/been involved in as a part of public involvement; and c) lessons learnt from this, including anything that worked well or did not work. Ten individuals (composed of researchers and PPI coordinators) joined the discussion, along with five members of the project team. A snapshot of the online discussions can be found in Fig. 5.

Participants on the Twitter chat felt that using arts-based approaches facilitated connections with audiences that might otherwise be difficult to engage in research. A few also felt that arts could be used to engage with diverse audiences on sensitive topics, as it can ease people into discussions and reduce awkwardness. It was suggested that arts can help develop ‘metaphors’ to describe complex scenarios or processes. Furthermore, the arts can capture, unpick and identify areas of concern which may otherwise be unexplored through other approaches. Overall, there was agreement amongst participants that arts in involvement activities enhanced discussions, addressed power imbalances and facilitated further reflections. Participants noted other interactive approaches to involvement including storytelling, collage making (wherein participants can choose the materials used) and drama.

### How we intend to use what we learnt from this project

We are members of the Evidence Synthesis Working Group (ESWG) (www.spcr.nihr.ac.uk/eswg), a collaboration of health services researchers and clinical academics from several universities in England, with an interest in a range of ways to synthesise evidence. We will use the artwork to propose topics for reviews to ESWG. We plan to return to the three groups involved in this project for PPI feedback on any review proposal that is taken forward based on priorities they identified. Those in these groups may also wish to be involved in the conduct and dissemination of findings from a related review (as PPI contributors). Other reviews we have conducted have involved patients/the public [18, 19]; such involvement was successful in helping us to think about data emerging from a review and in providing us with feedback on the presentation and interpretation of its findings.

## Discussion

We have shown the feasibility of using art to engage with potential PPI contributors who might not ordinarily be part of the planning or execution of research. We believe that the approach we used offered people the opportunity to undertake PPI in a relatively relaxed environment, by having a focus of producing some artwork as an output. Comments from attendees suggested they found this enjoyable and welcomed the production of something tangible from their input – the artwork – alongside knowing that they had contributed to research priority setting. For researchers, it was gratifying to work with the PPI contributors in this way, which we felt provided an informal and enjoyable background to knowledge generation. We hope that it will benefit healthcare delivery by identifying issues of priority to individuals who have traditionally been seldom listened to when it comes to PPI and research priority setting.

PPI in research is often limited to established /“go to” research user groups associated with universities and research centres. Our project contributors were part of community-based groups that had not been involved previously in PPI. They brought fresh perspectives to health concerns affecting them and expressed research priorities based on their lived experiences. Drivers for incorporating a range of voices in shaping research in this way include those that could be classed as moral (e.g. ensuring that research addresses issues of importance to patients, informing how public funding is spent) and practical or methodological - ensuring that quality research is produced (e.g. helping with recruitment through connections with specific communities, designing a study in a way that is acceptable to those who might take part, disseminating beyond the academic community so that findings have broader impact) [20, 21]. As noted by Gove and colleagues [1]:

*“Researchers have a legal and moral obligation to protect not only participants, but everyone involved in the research process from harm whilst striving to ensure that the process and outputs of PPI are successful, meaningful and mutually beneficial.”*

However, research does not always involve PPI contributors in a manner that embraces these moral and practical aspects, and there may be certain groups of people whose voices are seldom listened to. We have highlighted that art may be one means of accessing what matters to them in terms of research prioritisation. We still need to assess whether packaging research priorities as a piece of art means that researchers draw on this knowledge when planning a project. More broadly, it is important to consider whether it informs their thinking about incorporating PPI into the design and delivery of a study. Our arts-based project presents a novel approach to how PPI can be carried out. It also highlights the potential for new outputs (i.e. artwork). This may encourage a wider range of individuals to get involved in PPI activities. However, some researchers may not see the value and feasibility of PPI [22, 23]. Furthermore, researchers may be disinclined to try a novel approach if they feel daunted by the prospect of embedding PPI into their work, especially when resources to undertake this activity in a meaningful manner are lacking [24].

On a more positive note, hearing from colleagues about their experiences of novel approaches to PPI may be beneficial [24]. We hope that the reflections we present in this paper will prompt further attempts by researchers to try something similar. Others have applied different art forms as part of the research process, as reflected in the Twitter chat mentioned above. For example, colleagues from ESWG used drama and actors to share their findings from a review on delegated home visits in primary care with an audience [25]. This highlights that creative approaches to support engagement and dissemination are possible. In order to be inclusive when considering PPI, a shift may be required in research centres and organisations whereby researchers are encouraged to move beyond traditional approaches and to think of novel ways of involving patients and the public.

Our project sought to explore an approach to involving a diverse range of people with research prioritisation, using art as a mechanism. We were not able (nor was it our intention) to test this approach with every type of group that might be defined as seldom heard or marginalised. We have shown that the approach we adopted can work, as well as highlighting what we might have done differently so that others can use our learning in their own PPI activities. There are a range of other groups that could be classed as seldom listened to as part of PPI [11] that we did not cover in this project. We would be pleased to see other researchers adopt and adapt the approach we have described above when considering involving these other groups.

## Conclusion

We worked with groups who are seldom listened to when it comes to PPI activities. We anticipated that a focus on artwork would make involvement and the information we produced accessible to a wide range of individuals. The approach we used proved successful in encouraging people to share their views and to see these represented visually. It highlights the potential of this approach to make PPI more appealing to those not familiar with it. For the project team, it enabled us to understand concerns and research priorities from the perspective of people with views that may have been overlooked previously when planning a study. We would use this approach again; lessons learnt would make it easier next time around. We have provided recommendations for others who might try a similar approach in the future. Furthermore, we hope the paper will stimulate discussions around novel approaches to PPI.

## Data Availability

Not applicable as this was not a research study. We have shared the artwork developed from the workshops.

## Abbreviation

PPI: Patient and Public Involvement
